# Research progress on pathophysiologic mechanisms, clinical treatment and predictive biomarkers in bronchopulmonary dysplasia: from the perspective of oxidative stress

**DOI:** 10.3389/fped.2024.1343870

**Published:** 2025-03-27

**Authors:** Min Li, Wen-Xiang Cheng, Shuo Li, Jing Wang, Yan-Rui Chen, Liang Li, Gui Yang

**Affiliations:** ^1^ Department of Otolaryngology, Longgang Central Hospital, Shenzhen, China; ^2^ Shenzhen Institutes of Advanced Technology, Chinese Academy of Sciences, Shenzhen, China; ^3^ Guangxi Hospital Division of the First Affiliated Hospital, Sun Yat-sen University, Nanning, China; ^4^ Department of Otolaryngology, Huazhong University of Science and Technology Union Shenzhen Hospital, Shenzhen, China; ^5^ School of Medicine, Southern University of Science and Technology, Shenzhen, China; ^6^ Department of Otolaryngology & Allergy Center, The Central Hospital of Shenzhen Longgang District, Shenzhen, China

**Keywords:** bronchopulmonary dysplasia, hyperoxia exposure, oxidative stress, antioxidant treatments, predictive biomarkers

## Abstract

With the global rise in preterm birth rates, bronchopulmonary dysplasia (BPD) continues to be a significant problem, affecting morbidity and mortality in surviving preterm infants. Preterm infants are particularly susceptible to oxidative stress induced by sudden increases in oxygen concentration, which plays a crucial role in the pathogenesis of BPD. Herein, we addressed the pathophysiologic mechanisms, clinical treatment, and predictive biomarkers of BPD from an oxidative stress perspective. We first review the importance of oxygen in preterm infants and point out that sustained exposure to hyperoxia exacerbates the susceptibility of the immature lung to free radicals. The antioxidant properties of clinical therapies for BPD in preterm infants are then summarized. Subsequently, based on lipid, protein, and DNA damage mechanisms, we obtained the most comprehensive, accurate, and representative oxidative stress biomarkers. A total of 37 research papers on oxidative stress in BPD were collected. We conclude that 8-OHdG is the most promising biomarker for early prediction of BPD pathogenesis compared to lipid and protein oxidative stress biomarkers.

## 1 Introduction

Preterm birth, defined as birth before 37 weeks of gestation, is a significant global health issue for infants (1). According to the World Health Organization's report, an estimated 15 million premature births occur annually worldwide, with preterm rates varying from 5% to 18% across countries in 2022. In 2019, preterm mortality accounted for approximately 47% of all under-5 deaths globally. The increasing incidence of preterm births has attracted considerable attention, becoming a focal point in medical research (1, 2). Several factors contribute to this trend, including changes in maternal age, with both very young and older mothers being at risk, as well as multiple pregnancies and high cesarean rates due to medical interventions. China, the second-most populous country in the world, contributes the second-highest number of preterm births, with more than 1 million births annually (3). Data from the Lancet Global Health indicates that the increasing preterm birth rate in China has accelerated since the adjustment of child policy. Two primary factors contributing to this surge include the rising maternal age at childbearing and the greater use of assisted reproductive technology, both of which elevate the multiple pregnancy rate and the proportion of late preterm births (4). Furthermore, infections caused by pathogens, chronic health conditions, inadequate prenatal care, socioeconomic disparities, and lifestyle factors such as smoking and substance abuse all play significant roles (5). Numerous clinical studies (6–8) and systematic reviews (9–12) have demonstrated the role of COVID-19 infection during pregnancy as a significant risk factor for adverse maternal and fetal outcomes, particularly preterm birth (5). These events indicate the urgent need for global attention to address the rising rates of preterm birth (13).

Premature neonates often struggle to adapt to the extrauterine environment due to their underdeveloped organs, making them vulnerable to various diseases, with BPD being particularly notable (14–16). It is noteworthy that approximately 80% of preterm infants aged 22–24 weeks and around 20% of those aged 28 weeks are affected by BPD (17). Despite advancements in perinatal medicine that have improved survival rates among premature infants, the incidence of BPD remains steady or even increases due to the rise in preterm births with underdeveloped lungs (13, 18, 19). Additionally, BPD, being a systemic condition, significantly impacts health and quality of life, leading to poor pulmonary function outcomes, an increased risk of requiring home oxygen therapy, and hospitalization for respiratory infections (20). Patients with BPD often experience airflow limitations, reduced gas transfer, and decreased lung density, which heighten their susceptibility to long-term chronic obstructive pulmonary disease (21, 22). Due to its high morbidity rates, diverse phenotypic variations, and substantial medical and economic burdens on healthcare systems, BPD has emerged as a prevalent and complex concern in perinatal medicine (17, 23–26).

Oxidative stress has long been recognized as a significant contributor to the development of numerous neonatal diseases, including BPD. Factors contributing to developing BPD-related oxidative stress include abrupt changes in postnatal oxygen tension, additional exposure to high oxygen levels due to respiratory insufficiency, deficient antioxidant mechanisms, infection, and inflammation. These factors increase oxidative burden, leading to lung injury and developmental abnormalities (27–31). Multiple studies have demonstrated elevated levels of oxidative stress biomarkers in newborns who develop BPD compared to those unaffected by the condition (32–36).

Clinical interventions for BPD encompass protective ventilation, pulmonary surfactants, steroids, caffeine, vitamin A, nitric oxide, and nutritional optimization (28). However, the efficacy and safety of some of these approaches remain controversial (31). Considering the theoretical potential of antioxidant therapy in mitigating oxidative stress and pulmonary injury in BPD (28, 31), this review aims to analyze these interventions from an antioxidant perspective to enhance their clinical applicability. Furthermore, research indicates an elevation in oxidative stress biomarkers even in the early stages of BPD, suggesting exposure to the detrimental effects of oxidative stress in immature lungs before developing BPD (37–40). Thus, early oxidative stress levels are critical for predicting BPD, aiding in disease severity assessment, and providing personalized precision therapies for affected preterm infants (27). Consequently, this review synthesizes the most accurate and representative oxidative stress biomarkers based on lipid, protein, and DNA damage mechanisms, discussing their utility in predicting BPD development.

## 2 The pathogenesis of BPD: oxidative stress in immature lungs

Reactive oxygen species (ROS) are highly reactive oxidative molecules or ions primarily generated by endogenous oxidative metabolism processes. Moderate ROS production is crucial in cellular signaling transduction, apoptosis, proliferation, and inflammation within biological processes. However, excessive ROS levels or compromised antioxidant defense mechanisms can lead to oxidative stress, resulting in cellular structural and functional damage, consequently contributing to various diseases (41–43).

Postnatal risk factors such as high oxygen levels, hypoxia, ventilation, infections, and inflammation dramatically increase ROS production and contribute to BPD (28, 29, 41–43). Mechanical ventilation leads to alveolar overinflation and damage and triggers the activation of inflammatory signaling pathways and pulmonary fibrosis, a critical factor in developing classical BPD (27, 44). With the introduction of antenatal steroids, exogenous surfactant therapy, and protective ventilation strategies such as reduced tidal volumes and decreased invasive ventilation, these clinical interventions have somewhat reduced the incidence and mortality rates of oxidative stress and BPD (31, 45).

Advancements in healthcare have shifted the gestational age criteria for BPD diagnosis from extremely premature infants (gestational age less than 32 weeks) to very premature infants (gestational age less than 28 weeks) (44), exposing the lungs to high oxygen levels during the late canalicular or early saccular stages (17, 46). Premature birth transitions the lungs from the low-oxygen environment of the uterus to the high-oxygen environment of indoor air, exposing them to relatively high oxygen concentrations (30). Fetuses *in utero* only need to cope with blood oxygen tensions of 25–30 mmHg and benefit from maternal antioxidant protection, thereby avoiding oxidative stress (47). In comparison, preterm infants must cope with oxygen tensions of 60–100 mmHg for an extended period with immature lungs. Furthermore, the alveolar gas exchange surface area at these stages is incompletely developed, unable to provide sufficient oxygen for metabolism (48, 49). Oxygen therapy has become a necessary standard treatment to prevent newborns from dying due to respiratory failure (13). These factors make infants prone to oxidative stress due to exposure to high oxygen concentrations, leading to the development of BPD. Notably, the endogenous antioxidant system within immature lungs fully matures shortly before full-term delivery (41). Consequently, the excess ROS cannot be effectively eliminated, leading to ROS accumulation and oxidative stress. Excessive ROS induces apoptosis and dysfunction of alveolar epithelial type II cells responsible for synthesizing and secreting pulmonary surfactant (29), impairing lung vascular and alveolar development (28), thus contributing to the emergence of the new phenotype of BPD (50, 51).

## 3 Clinical treatment of BPD: an antioxidant perspective

The treatment landscape for BPD continues to evolve, yet achieving satisfactory outcomes remains elusive (17, 52, 53). Both basic researchers and clinicians are challenged by the iatrogenic injuries associated with improving the survival of preterm infants (54). Given the central role of oxidative stress in BPD pathogenesis, strategies targeting antioxidants hold promise for prevention and treatment (42, 55). Numerous studies on antioxidant therapy and reviews summarize the clinical treatment methods (56–60). However, the antioxidant aspects of the clinical treatment of BPD remain essential. Therefore, this article reviews current clinical approaches to BPD treatment from an antioxidant perspective.

We searched the PubMed database for published clinical studies covering the neonatal (birth-1 month) up to 2023. The search strategy included medical subject headings (MeSH headings) and free text terms related to antioxidants, pulmonary surfactants, vitamin A, vitamin E, vitamin D, caffeine, nutritional interventions, and BPD.

### 3.1 Pulmonary surfactant

Since the certified importance of pulmonary surfactants in enhancing the survival of preterm infants (61), the administration of prenatal corticosteroids to boost endogenous surfactant production before birth and the introduction of exogenous surfactants after birth have been pivotal milestones in neonatal medicine (62). These developments have altered the original definition of BPD proposed by Northway in 1967 and launched the post-surfactant era (20, 63).

Pulmonary surfactants coat the alveolar surface and are complex mixtures of phospholipids and proteins (64, 65). The alveoli of preterm infants are at the stage of the late canalicular or early saccular periods during which type II alveolar cells have not fully developed, leading to alterations in the quantity, quality, or composition of surfactant secretion (66). Additionally, surfactant proteins are influenced by hyperoxia, potentially prolonging the need for ventilator support and increasing the risk of BPD (67–69). Studies in preterm infants have demonstrated that natural surfactant treatment reduces oxidative stress parameters in tracheal aspirates from ventilated infants (25, 70–72). Firstly, exogenous surfactant therapy can improve neonatal adverse outcomes by reducing inhaled oxygen concentration and exogenous ROS formation during oxygen therapy (68, 73). Secondly, surfactant proteins inhibit inflammatory processes and enhance microbial clearance (66), which also play a vital role in reducing the production of endogenous ROS to some extent (74). Thirdly, natural surfactants contain polyunsaturated phospholipids along with enzymatic antioxidants like SOD and CAT (75–77), thereby shielding the cell membrane from the assault and damage caused by ROS. These properties also underscore the susceptibility of lungs with an insufficient antioxidant system to oxidative damage under hyperoxia (78).

Since the beginning of the last century, natural surfactants have been regarded as superior to synthetic surfactants in enhancing respiration, reducing mortality, and lowering the incidence of BPD (79, 80). Treatment with poractant alfa may offer more advantages than calfactant and other animal-derived surfactants in preventing BPD (81, 82), potentially due to its lower dipalmitoyl phosphatidylcholine value and higher activity of antioxidant components both in natural surfactants and poractants (83). Additionally, the differentiation of epithelial type II alveolar cells and the surfactant production rate may be regulated by endogenous glucocorticoids and accelerated by exogenous glucocorticoids, primarily through the regulation of gene expression associated with increased surfactant protein synthesis, which has been extensively reported in animal experiments (75, 84–87). Although antenatal corticosteroids are commonly regarded as a routine approach to promoting fetal lung maturation in preterm birth, there remains controversy surrounding their postnatal use for preventing BPD, including issues related to the timing of administration, choice of agents, and routes of administration (88, 89).

### 3.2 Vitamin A

Vitamin A, the best-studied non-enzyme antioxidant in BPD, plays a crucial role in regulating fetal lung development and maturation, maintaining the integrity of respiratory epithelial cells, influencing pulmonary vessel development, reducing the need for supplemental oxygen in premature infants, and ultimately decreasing premature infant mortality (25, 90). Many investigations suggest that vitamin A deficiency is prevalent in very-low-birth-weight infants from birth to term (91, 92). The deficiency in preterm infants may be related to the maternal vitamin A level or inefficient placental transmission, which leaves them malnourished (93, 94). Vitamin A deficiency is also associated with BPD, and there has been considerable evidence that supplementation can reduce the mortality of BPD and infant mortality (95–97).

In terms of sources, the two primary forms of vitamin A in our bodies are animal-derived (including retinol and its derivatives) and plant-derived carotenoids (such as α-carotene, β-carotene, and β-cryptoxanthin) (25, 98). As retinol is the main active form of vitamin A in the human body and has high absorption, the types of vitamin A used in clinical practice are retinol and its derivatives (98). Animal-derived vitamin A can act as a chain-breaking antioxidant, preventing cell damage by inhibiting the interaction of peroxyl radicals with lipids to produce hydroperoxides (99, 100). Despite the lower absorption and conversion rates compared to retinol, it is still worth exploring whether carotenoids can reduce the incidence of BPD, primarily owing to their direct antioxidant activities in scavenging singlet oxygen and peroxide-free radicals (101, 102), as well as increasing the production of enzymatic antioxidants (103, 104).

Currently, research on the delivery route of vitamin A primarily focuses on intramuscular and oral administration. While intramuscular administration can effectively reduce BPD and infant mortality rates, it is costly and associated with painful side effects (105, 106). The efficacy of oral administration in reducing BPD incidence remains debated. Additionally, intratracheal administration shows promise due to its ability to increase retinol concentration in tissues like serum, liver, and lungs in animal models, indicating its feasibility and warranting further investigation (107–109). Although the preventive effect of vitamin A on BPD is still controversial, it has become more widely used as a result of the increasing rate of very-low-birth-weight infants (18, 110–112).

### 3.3 Vitamin E

Like vitamin A, vitamin E is also a crucial fat-soluble non-enzymatic antioxidant with potent anti-inflammatory and antioxidant properties, playing a significant role in embryonic lung development (113, 114). Research demonstrates that the fetus primarily obtains vitamin E from the mother through the placenta (115). Fetal levels of vitamin E are closely correlated with maternal levels during the same period and increase with gestational age (116, 117). Given our inability to synthesize this nutrient, premature and low birth weight infants commonly experience vitamin E deficiency, increasing the risk of adverse outcomes such as BPD (115, 118, 119). Moreover, premature infants have an increased demand for non-enzymatic antioxidants compared to full-term infants. Hence, additional vitamin supplementation may lower the incidence of BPD (117, 120, 121). However, research on the effectiveness of vitamin E is limited in duration, quantity, and depth when compared to vitamin A. Most studies on oral and intravenous administration of vitamin E have failed to demonstrate a reduction in BPD incidence and have even led to adverse outcomes such as infant death and necrotizing enterocolitis (122–124). Consequently, vitamin E supplementation was temporarily suspended in the late 1990s.

More recently, Ogihara and Mino discussed the research findings on BPD conducted during the pre-surfactant era in the 1980s and early 1990s (115). They suggested that these findings may not apply to modern neonatal care due to significant differences in the definition of prematurity between the pre-surfactant and post-surfactant eras (25, 55, 115). Since 2000, studies have re-confirmed the relationship between vitamin E deficiency and the severity of BPD (125, 126), although progress remains slow and the outcomes are preliminary (127–129). Since vitamin E is an essential component of pulmonary surfactant and plays a crucial role in the post-surfactant era (119, 130, 131), it is unsurprising that vitamin E possesses anti-inflammatory and antioxidant properties that can mitigate the incidence of BPD. In addition, high levels of vitamin E have been detected in breast milk, indicating its essentiality for newborns with specific nutritional needs, especially in low birth weight infants (118, 132, 133). Although various isomers of vitamin E have similar antioxidant functions in preventing lipid peroxidation (114, 134), high doses of α-tocopherol were widely used due to its availability in the human body during the pre-surfactant era (117, 135). Data on the effectiveness of specific subtypes and different routes of administration for vitamin E supplementation trials on lung health are necessary to determine whether re-challenging in modern neonatal intensive care units is worthwhile (114, 115, 130), which may improve lung development and can reduce the adverse outcomes caused by the injection and oral administration.

### 3.4 Vitamin D

Since 2000, there has been an increase in studies examining the relationship between vitamin D deficiency and BPD. Apart from its various immunomodulatory and anti-inflammatory functions (136), it possesses indirect antioxidant properties that may alleviate conditions induced by oxidative stress, such as diabetic retinopathy (137), endothelial dysfunction (138), skin aging (139), and mood disorders (140). The potential mechanism for reducing oxidative stress involves vitamin D enhancing the expression of SOD2, glutathione, and nuclear factor NRF2, which are responsible for antioxidant enzyme expression (137–139, 141).

The vitamin D level of early neonates is closely linked to that of their mothers and gestational age, as the fetus obtains vitamin D from the mother via the placenta (142). As a result of modern lifestyles, vitamin D deficiency is prevalent in pregnant women (143), impacting serum vitamin D levels in preterm infants and subsequently impairing lung development (144). Several clinical studies and reviews have established an association between neonatal BPD and low vitamin D levels, with a higher incidence observed in the vitamin D deficiency group (127, 144–146). A low level of 25-hydroxyvitamin D in the bloodstream is a valuable predictor for the prediction of BPD (147–149). Although early vitamin D supplementation has shown promise in significantly increasing serum levels of 25(OH)D3, reducing inflammatory responses, and decreasing the incidence of BPD in preterm infants, more extensive clinical trials with varying doses are still necessary (150–154).

Recent research has demonstrated that vitamin D administration improves alveolar structural simplification induced by hyperoxia and elucidated underlying mechanisms from the perspective of reducing inflammation (155–161). Vitamin D administration reduced the expression of proinflammatory cytokines IL-6, IFN-γ, TNF-α and IFN-γ (156–158), regulated the balance of M1 and M2 macrophages by decreasing the expression of IL-10 and Arg-1 (155), protected neonatal rats from hyperoxia-induced BPD by regulating the vitamin D-VDR signaling pathway (158), antagonized the activation of TLR4 (159), contributed to the recovery of mitochondrial morphology (156), reduced cell apoptosis (156, 159), and promoted the growth of vascular structures (157). In addition, low doses of vitamin D improve the formation of alveolar and pulmonary vascularization in BPD by inhibiting neutrophil extracellular traps under hyperoxia, whereas higher doses may lead to more severe outcomes (157). Moreover, inhaled vitamin D is crucial for promoting surfactant phospholipid synthesis, which is vital in reducing oxidative stress caused by hyperoxia exposure (161). Overall, due to its potent antioxidant properties and the potential benefits of inhalation over oral administration in promoting neonatal lung development (137, 161), further studies on vitamin D supplementation from an antioxidant perspective are warranted to reduce the incidence of BPD.

### 3.5 Caffeine

Caffeine, a methylxanthine drug, has been widely used to treat apnea of prematurity for decades and is one of the few pharmacological interventions that has been shown to significantly reduce the risk of BPD in preterm infants (162). Caffeine treatment primarily enhances diaphragmatic contractility, increases minute ventilation, and stimulates the central nervous system, effectively improving respiratory function in preterm infants. These mechanisms help reduce the need for mechanical ventilation, improve lung function, and facilitate successful extubation (163). Therefore, caffeine therapy plays a crucial role in lowering the incidence of BPD. Multiple clinical trials have supported the clinical benefits of caffeine in treating and preventing BPD (163). As a result, current research on caffeine therapy mainly focuses on the timing of treatment and dosage. Although increasing evidence supports the early use of caffeine in preterm neonates, formal guidelines specifying the exact timing to start treatment have yet to be established. Clinical trials are needed to determine the optimal timing for caffeine administration and to identify the infant population that would benefit most from early caffeine therapy. Further studies are also necessary to validate and elucidate the precise impact of early caffeine treatment on complications in preterm infants.

Yan et al. found that early administration of caffeine can reduce the severity of BPD by approximately 60% (164). Similarly, Chen et al. reported that caffeine administration within the first three days of life shows promising results in preventing severe BPD and mortality in extremely preterm infants (165). Other studies have indicated that early preventive use of caffeine citrate not only significantly reduces the incidence of BPD in preterm infants but also decreases the occurrence of other complications. Ye et al. found that early preventive administration of caffeine citrate reduced the risk of later free radical disease in preterm infants, including BPD (166). Jiang et al. found that early preventive use of caffeine citrate is more effective than standard caffeine treatment in reducing the incidence of BPD in preterm infants (167). Szatkowski et al. reported that an increased proportion of early preventive caffeine use is associated with a reduced risk of BPD and brain injury in preterm infants (168). By comparing early preventive use of caffeine citrate (within 72 h after birth) and standard caffeine treatment, Elmowafi et al. found that the former reduces the duration of oxygen therapy, ventilation needs, and the incidence of mild to moderate BPD in preterm infants (169). Lamba et al. found that early high-dose caffeine therapy (10 mg/kg/day) lowers the risk of moderate to severe BPD without increasing the incidence of measured complications (170). Rauf et al. observed that early initiation of high-dose caffeine can prevent apnea and extubation failure in preterm neonates (171). Additionally, some studies suggest that early caffeine treatment may lead to complications and even increase mortality rates. Taha et al. found that early preventive use of caffeine citrate improves survival rates in preterm infants without BPD. However, it also increases the risk of fatal necrotizing enterocolitis (172). Similarly, research by Dobson (173) and Yun (174) indicates that while early oral caffeine treatment reduces the incidence of BPD, it is accompanied by an increased mortality rate.

Evidence suggests that caffeine and its methylxanthine metabolites may reduce oxidative stress by modulating inflammation-related pathways. Caffeine inhibits oxidative stress by suppressing the activation of IRE1 and PERK induced by endoplasmic reticulum stress to prevent skin senescence (175) or by activating A2AR/SIRT3/AMPK-mediated autophagy in the leptin-induced phosphorylation of STAT3 (176). Caffeine treatment has also been shown to reduce oxidative stress by enhancing the activity of antioxidant defense enzymes, mitigating DNA damage, and modulating transcription, indicating its antioxidant function to some extent (23). Recent clinical evidence indicates that caffeine's protective effect on neonatal lung health might involve reducing the expression of genes such as MMP9, TNF-α and TLR4, thus alleviating pulmonary inflammation which may be a mechanism behind the significant reduction in BPD incidence observed in the caffeine treatment group (167). Furthermore, although there is no statistically significant difference in BPD incidence between preventive and treatment groups, the preventive group showed significantly lower levels of IL-6 and IL-8 than the treatment group (177). This reduction in cytokine levels may contribute to a lower incidence of BPD.

Although clinical studies have not yet clarified how caffeine reduces the incidence of BPD through mediating redox pathways (178), a series of cellular and animal experiments indicate that caffeine has antioxidant properties. In a cellular model of BPD induced by hyperoxia, caffeine may reduce apoptosis, promote proliferation, and alleviate oxidative stress by inhibiting the A2AR/cAMP/PKA/Src/ERK1/p38MAPK signaling pathway, thus preventing lung damage (179). In the animal model of BPD, caffeine treatment significantly mitigates cell death and changes in apoptosis-related factors induced by hyperoxia (180) and protects murine lungs from oxidative damage by inhibiting the NLRP3 inflammasome and NF-κB pathways, which reduces apoptosis in type II alveolar epithelial cells (181). Additionally, caffeine treatment may protect developing lungs from injury induced by hyperoxia by alleviating endoplasmic reticulum stress (182).

Given caffeine's important role in the prevention and treatment of BPD, there is still a lack of comprehensive understanding of its molecular mechanisms, particularly regarding whether caffeine treatment can reduce lung injury by alleviating oxidative stress. Therefore, further research in this area is essential.

### 3.6 Nutritional interventions

Premature infants fail to regulate inflammatory immune responses, resulting in sustained lung injury and chronic pulmonary inflammation (183). However, specific functional nutrients with antioxidant properties may play a role in reducing pulmonary inflammation (184). The primary consideration here is the potential mechanism of breast milk as an antioxidant therapy, as single antioxidant therapy for BPD has not yielded the expected clinical outcomes (28). Yang et al. indicated that breast milk is the safest, most natural, and most comprehensive infant nutrition (28). It provides all the necessary calories, proteins, and lipids for newborn growth and development, along with various antioxidants such as unsaturated fatty acids, vitamins, trace elements, glutathione (GSH), SOD, glutathione peroxidase (GSH-Px), melatonin, probiotics, short-chain fatty acids, and lactoferrin, offering robust antioxidant capabilities to newborns (28, 185).

In clinical practice, breastfeeding has been shown to reduce the incidence of BPD in premature infants significantly. Compared to formula feeding, both exclusive breastfeeding and pasteurized donor human milk feeding have resulted in a lower incidence of BPD in premature infants (186, 187). A higher incidence of BPD was also found in preterm infants who received pasteurized or frozen breastfeeding compared to exclusive breastfeeding (188, 189). The possible reasons are as follows. Although carbohydrates remain relatively intact, many components in breast milk change during freezing, pasteurization, and subsequent reheating. Freezing breast milk increases the formation of lipid peroxides, which can damage cell membranes, increase oxidative stress, and potentially lead to cellular injury. Furthermore, freezing decreases the concentration of bioactive proteins, such as secretory immunoglobulin A, lactoperoxidase, and lysozyme in breast milk, which is crucial in combating oxidative stress and maintaining immune balance. Moreover, the processes may reduce the content of antioxidants in breast milk and lead to the loss of activity of immune cells and stem cells, which are vital for protecting infants from oxidative damage and promoting tissue repair (186–189).

The amount of breast milk intake in premature infants is negatively correlated with the incidence of BPD (190). For premature infants breastfed from birth to 36 weeks postmenstrual age, each 10% increase in breastfeeding was accompanied by a 9.5% reduction in the risk of developing BPD (191). Based on these studies, breast milk seems beneficial in preventing and treating BPD. Given the various antioxidants in breast milk, we believe this is a primary mechanism by which breast milk helps reduce BPD (28).

Currently, it remains uncertain whether individual components of breast milk can function independently as antioxidants due to inconsistent findings in this area (28). For instance, taking Omega-3 polyunsaturated fatty acids as an example, the risk of BPD increases with decreased DHA levels and increased LA (192). The incidence of BPD was reduced in infants whose mothers received a DHA diet compared to those who did not (184). However, some studies have found that Omega-3 polyunsaturated fatty acid interventions do not affect the incidence of BPD (193, 194). Similar results are seen with trace elements, vitamins, and lactoferrin (28, 185). Possible reasons for this inconsistency include the limited efficacy of single-ingredient antioxidants for treating BPD.

### 3.7 Other antioxidant treatments

Among all antioxidant enzyme replacement therapies for preclinical strategies, recombinant human SOD has shown the most promising outcomes (25). Though no positive effect on mortality and morbidity was observed at 36 weeks postmenstrual age in the treatment of intratracheal recombinant human SOD, the number of infants with respiratory sequelae or requiring pulmonary resuscitation decreased at 1-year corrected age (195). A clinical trial has also investigated the role of recombinant human SOD therapy in alleviating ROP, a form of oxygen radical disease. The incidence of ROP was significantly reduced in infants younger than 25 weeks (196). Despite these advancements, progress in recombinant human SOD therapy has been slow over the years. In animal trials, overexpression of SOD at the transcriptional level (59, 60), oral supplementation of coenzyme Q10 (57), caffeine (95), and chrysin treatment (197) could alleviate lipid oxidative stress, decrease alveolar damage, and improve lung function. Research has shown that glucosinolate and quercetin can reduce lung inflammation by regulating transcription factors or antioxidant-related proteins (56, 58). Although antioxidant treatments have shown efficacy through histopathological assessment of target organs in many animal models, translating basic research findings into clinical practice remains challenging (198, 199).

## 4 Oxidative stress-related biomarkers

The previous chapter described possible mechanisms for reducing oxidative stress through treating BPD in the clinic. Currently, there is a lack of studies linking oxidative damage to outcomes. However, there is a strong need to identify validated biomarkers of oxidative stress, which would provide a theoretical basis for clinicians to develop preventive and immediate adjustments to therapeutic strategies, improve the prognosis of preterm infants, and reduce the burden of this condition on the preterm infant. Although biomarkers of oxidative stress have been identified in different tissues or body fluids, many of these biomarkers do not correlate well with BPD, do not reflect the state of oxidative stress, or lack specificity. Therefore, the focus should be on understanding which markers can be practically applied in the clinic to predict the occurrence of BPD.

Overall, oxidative stress can be categorized into four broad categories: “antioxidant defenses”, “lipid peroxidation”, “nucleic acid oxidative damage”, and “oxidative damage to proteins”. The antioxidant defense system consists of enzymatic and non-enzymatic categories. The former mainly include superoxide dismutase (SOD), catalase (CAT), glutathione peroxidase (GPx), thioredoxin reductase (TRX), peroxiredoxin (PRX), and glutathione S-transferase (GST), while the latter mainly includes metal-binding proteins (MBP), ascorbate (AA), Vitamin E, Vitamin C, uric acid, and GSH (200).

### 4.1 Oxidative stress-induced lipid peroxidation and corresponding biomarkers

Structural lipids, also known as membrane lipids, are essential components of cells, such as the plasma membrane, Golgi apparatus, and endoplasmic reticulum. They are composed of more than 70% phospholipids and 10%–20% cholesterol (Ch). Each phospholipid consists of two esterified fatty acyl chains, one saturated (sn-1 chain) and the other unsaturated (sn-2 chain). The unsaturated chain includes linoleic acid (LA, C18:2, omega6), arachidonic acid (AA, C20:4, omega-6), conjugated linoleic acid (CLA, C18:2, omega6), eicosapentaenoic acid (EPA, C20:5, omega-3), docosahexaenoic acid (DHA, C22:6, omega-3), and alpha-linolenic acid (ALA, C18:3, w-6). Among these, LA, AA, DHA, and Ch are the most abundant unsaturated fatty acids in mammalian cell membranes, and their double bonds are susceptible to oxidation under oxidative stress, leading to lipid peroxidation (201).

**Figure 1:**
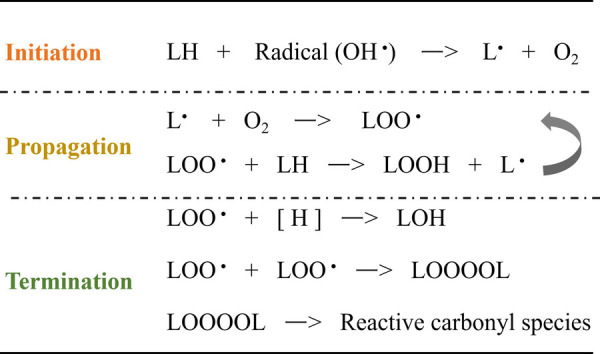
Schematic representation of the free radical-driven oxidative stress process for lipid.

Lipid peroxidation, driven by a free radical chain reaction, consists of three reaction phases: initiation, propagation, and termination (202, 203). In the initial phase, hydrogen atoms in the methylene groups of the unsaturated fatty acids side chains are captured by pro-oxidants such as hydroxyl radicals (OH^•^) to produce lipid radicals (L^•^). During the propagation phase, L^•^ reacts with O2 to form lipid peroxyl radicals (LOO^•^). LOO^•^ can further extract hydrogen atoms from neighboring PUFA residues to generate new L^•^ and lipid peroxyl radicals (LOOH), and the resulting new L^•^ drives lipid peroxidation similarly. In the termination phase, the LOO^•^ species reacts with hydrogen atoms from the antioxidant to generate lipid hydroxide (LOH) or combines with another LOO^•^ to form non-radical product, as shown in Figure 1.

**Figure 2:**
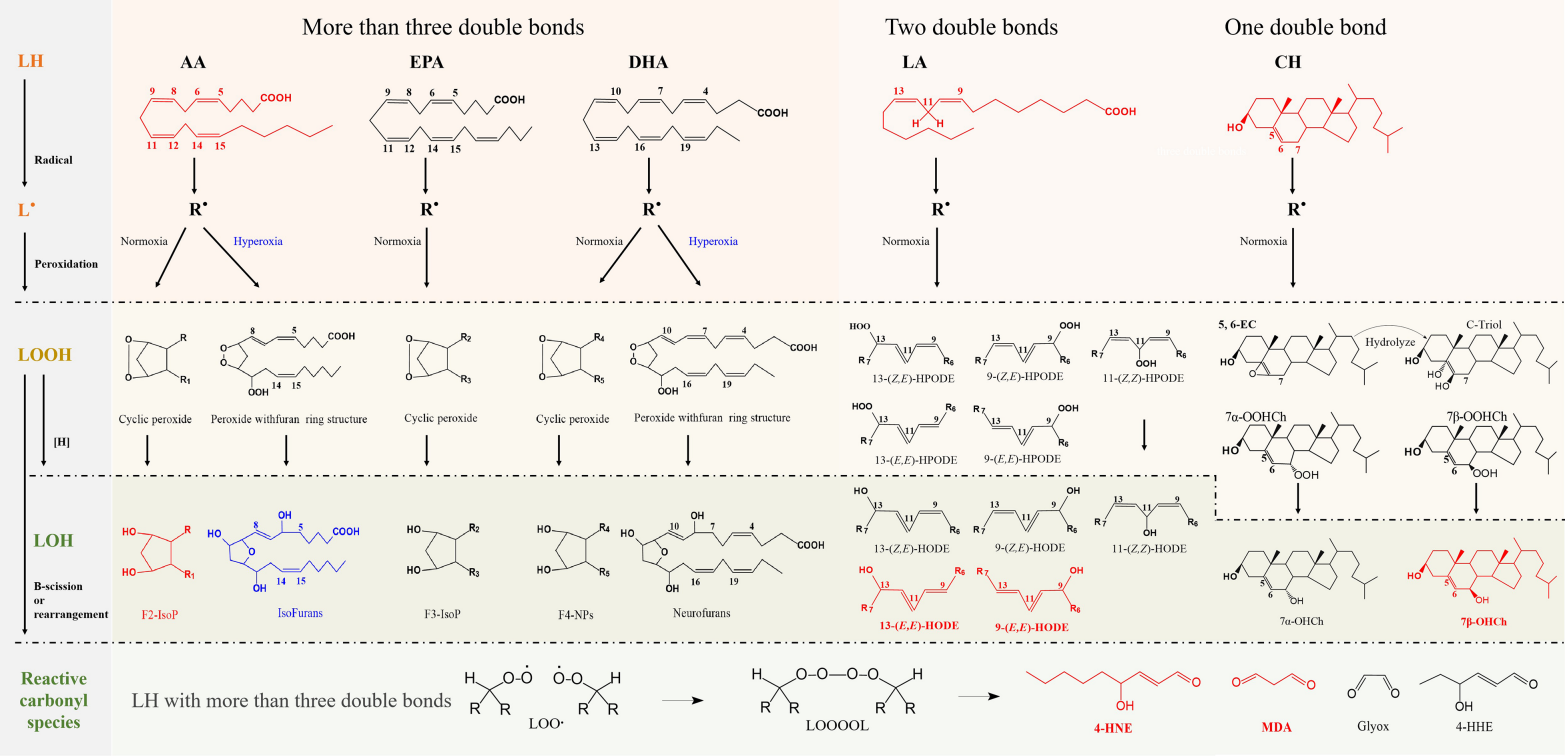
Schematic representation of the free radical-driven oxidative stress process for lipid. The processes in generating biomarkers for lipid peroxidation and the abbreviations of representative biomarkers are highlighted in red.

Unsaturated fatty acids with more than three double bonds generate intracyclic peroxide intermediate isomers (LOOH), which are subsequently reduced to prostaglandin-like compounds (LOH) and their isomers. This process involves the formation of F2-IsoPs from AA, F3-IsoPs from EPA, and F4-neuroprostanes (NPs) from DHA. These primary products are further metabolized by Hock rearrangement or β-breakage reactions to form toxic reactive carbonyl species (RCS), including malondialdehyde (MDA), 4-hydroxynonenal (4-HNE), formaldehyde (FA), acrolein, methylglyoxal (MGO) (202–204). The RCS readily triggers irreversible modification and cross-linking with proteins, nucleic acids, and other biological macromolecules, resulting in physical dysfunction, as shown in Figure 2.

In contrast, fatty acids containing fewer double bonds are less susceptible to oxidation than substances containing polyunsaturated fatty acids. Linoleates (containing two double bonds), the body's most abundant polyunsaturated fatty acids, and Ch (monounsaturated lipids) generate oxidized products only through free radical chain reactions. LA generates hydroperoxyoctadecadienoic acid (HPODE) and its isomers, and these hydroperoxides are subsequently reduced to hydroxyoctadecadienoic acid (HODE) and its isomers (204, 205). Ch generates 7α- and 7β-hydroperoxycholesterol (7α-OOHCh and 7β-OOHCh) as well as 5α, 6α- and 5β, 6β-epoxycholesterol. The first two are subsequently reduced to 7α- and 7β-hydroxycholesterol (7α-OHCh, 7β-OHCh), and the latter can be produced as Cholestane-3β,5α,6β-Triol (C-Triol) in the presence of hydrolases (206). However, 7α-OHCh can be generated by enzymatic reactions, and thus, 7β-OHCh is considered the primary product of Ch under free radical mediation, as shown in Figure 2.

Lipid hydroxides may serve as more suitable biomarkers than hydroperoxides, and trans-hydroxides are formed only due to free radical-mediated peroxidation (200, 202). Considering the abundance of unsaturated fatty acids, it is generally accepted that F2-IsoPs, HODE, and 7β-OHCh are the primary oxidative stress markers (highlighted in red), as shown in Figure 2. In addition, with increasing oxygen tension, isofurans (IsoFs) and neurofurans (NFs) can be formed by peroxidation of AA and DHA, respectively (204). The formation mechanism of IsoFs and NFs compounds is similar to that of F2-IsoPs, as shown in Figure 2.

### 4.2 Oxidative stress-induced protein oxidation and corresponding biomarkers

Free radicals attack protein molecules and mainly affect amino acid side chains, which causes them to undergo oxidative modifications. These modifications include oxidation of sulfur-containing amino acids, oxidation of aromatic amino acids, carbonylation, sugar oxidation, and nitration formation (207).

Although all amino acids can be modified by ROS, sulfur-containing amino acids (methionine, cysteine) are particularly susceptible to oxidative reactions due to the higher sensitivity of the sulfur group. Cysteine thiol undergoes two oxidation pathways (207). One pathway involves the reversible formation of a disulfide bond with another thiol, regulated by several intracellular enzymes. The second pathway involves the gradual oxidation of thiol, resulting in the reversible formation of sulfenic acid (-SOH) and sulfinic acid (-SO_2_H) or the irreversible formation of sulfonic acid (-SO_3_H) (208). The selenol of selenocysteine can be regarded as a specialized thiol as it catalyzes thiol/disulfide exchange reactions (209). Like thiols, selenols can undergo oxidation to diselenides, selenoxides, selenenic acids, seleninic acids, and selenonic acids (210, 211). Selenium's higher nucleophilicity and electrophilicity enable it to efficiently cycle between its reduced state (selenols) and oxidized state (diselenides), making it an effective redox regulator (212). Notably, five glutathione peroxidases (GPx), three thioredoxin reductases (TrxR), and methionine sulfoxide reductase 2 (MsrB) are selenium enzymes involved in redox reactions (213).

Methionine is highly susceptible to ROS oxidation, a reaction prevalent in almost all living organisms (214). Under more intense experimental conditions, such as higher concentrations of N-bromosuccinimide, methionine sulfoxide can undergo irreversible oxidation to methionine sulfone (215). However, relatively few studies have investigated the physiological conditions of methionine sulfone. In aging mice, methionine sulfoxide and methionine sulfone have been identified as the most abundant amino acid oxidation modifications. The levels of methionine sulfone in histones and the cytoplasm of aging mice are significantly higher than those in young mice (216). In contrast to the well-known susceptibility of cysteine to oxidation, methionine oxidation has been largely overlooked (217). This oversight can be attributed to its hydrophobic and relatively weak nucleophilicity in the thioether group of methionine (218). Additionally, the reversible catalysis of methionine sulfoxide by methionine sulfoxide reductases and its identification as methionine in traditional Edman sequencing procedures further contributes to the neglect of methionine oxidation (219). The levels of methionine sulfoxide indicate the overall cellular redox status, making it a promising clinical biomarker (207, 215).

The oxidation of other amino acids requires more stringent conditions than that of sulfur-containing amino acids, and aromatic amino acids are secondarily susceptible to oxidation because their molecular structure contains multiple conjugated double bonds, and their high electron density properties make them susceptible to oxidation, generating various stable oxidative modification products (220). Tyrosine generates an intermediate tyrosyl radical followed by dihydroxyphenylalanine or bis-tyrosine. Tryptophan is oxidized to hydroxytryptophan by hydroxyl radicals, followed by oxygen cleavage of hydroxytryptophan to form N-formyl kynurenine. Hydroxyl radicals oxidize phenylalanine and histidine to form ortho-tyrosine and 2-oxohistidine, respectively (207).

The oxidative modification of amino acids formed by the combined action of glycosylation and oxidation, known as glycoxidation, is an irreversible process (221). Reducing sugars first react non-enzymatically with free amino groups in amino acids or proteins (usually lysine or arginine) to form compounds with sugar groups called basal glycation products. The basal glycation products are susceptible to oxidative stress, which triggers oxidation and condensation reactions. These reactions lead to further structural changes in the basal glycation products to form more complex advanced glycation end products (AGEs). The most abundant AGEs in the body are carboxymethyl lysine and pentosidine, produced from lysine and formed by cross-linking between lysine and arginine residues, respectively (207).

Nitrification modifications are also formed under conditions of oxidative stress. Nitric oxide reacts with superoxide anion to form the reactive nitrogen species—peroxynitrite, which subsequently undergoes nitration with phenolic groups in the tyrosine molecule, irreversibly adding nitro substituents to the amino acid side chain of tyrosine to form 3-nitrotyrosine (207, 222).

The process of introducing reactive carbonyl groups such as aldehydes, ketones, and lactams into the amino acid side chains of proteins is called “protein carbonylation” (223). Protein carbonylation is usually defined as an irreversible post-translational modification that causes conformational changes in the polypeptide chain and results in loss of protein function. Carbonylation is relatively challenging to induce compared to other oxidative modifications and is considered a significant marker of oxidative damage to proteins. The protein carbonylation generation pathways in which ROS are directly involved fall into two categories. ROS can directly attack lysine, arginine, proline, and threonine side chains, introducing carbonyl groups and generating α-aminoadipic acid semialdehydes (AAS) derived from lysine and γ-glutamic acid semialdehydes derived from arginine and proline. In addition, the active carbonyls formed by ROS-attacking lipids can also be added to nucleophilic amino acids (i.e., cysteine, histidine, and lysine) by Michael addition or by generation of Schiff bases (207, 224).

These modifications also irreversibly cause proteins to produce cross-links, altering the composition and folding of proteins and affecting their function as receptors, enzymes, carriers, or structural proteins (207). Advanced oxidized protein products (AOPPs) are cross-linked protein-containing products containing dityrosine and carbonyl groups formed by the reaction of plasma proteins with oxidants and are also considered markers of oxidant-mediated protein damage (225).

### 4.3 Oxidative stress-induced nucleic acid oxidation and corresponding biomarkers

Due to the reactivity of nitrogen and oxygen atoms in nucleic bases, nucleic acids are highly susceptible to damage induced by oxidative stress. While all four bases are impacted by ROS, guanine (G) exhibits the lowest redox potential relative to the other bases (G: −3.0 V, A: −2.71 V, C: −2.56 V, and T: −2.32 V) (226). As a result, guanine nucleotides and deoxyguanine nucleotides are more prone to oxidation, resulting in the formation of 8-oxoGuo and 8-oxo-dG, respectively. Furthermore, 8-oxoGuo and 8-oxodG can be released into the bloodstream and excreted in urine, allowing their detection in human serum/plasma and urine samples. They serve as well-established biomarkers for oxidative damage to DNA and RNA (227, 228).

However, the oversight of RNA oxidative damage primarily stems from the relatively short half-life of RNA, and the initial research focused on DNA oxidation, leading previous studies to concentrate predominantly on DNA. In fact, RNA molecules are more susceptible to the influence of reactive oxygen species (ROS) than DNA. This susceptibility is mainly due to ribonucleotides being more abundant than deoxyribonucleotides, and RNA lacks protective and repair mechanisms, making its bases more prone to oxidation. Although previous research has linked 8-oxoGuo to various diseases, including Alzheimer's disease (229, 230), Parkinson's disease (231), type 2 diabetes (232), obesity (233), atherosclerosis (227), heart failure (234), and tumors (235, 236), studies on RNA oxidative stress products related to BPD, are yet to be reported. Hence, this section primarily discusses DNA oxidation.

**Figure 3:**
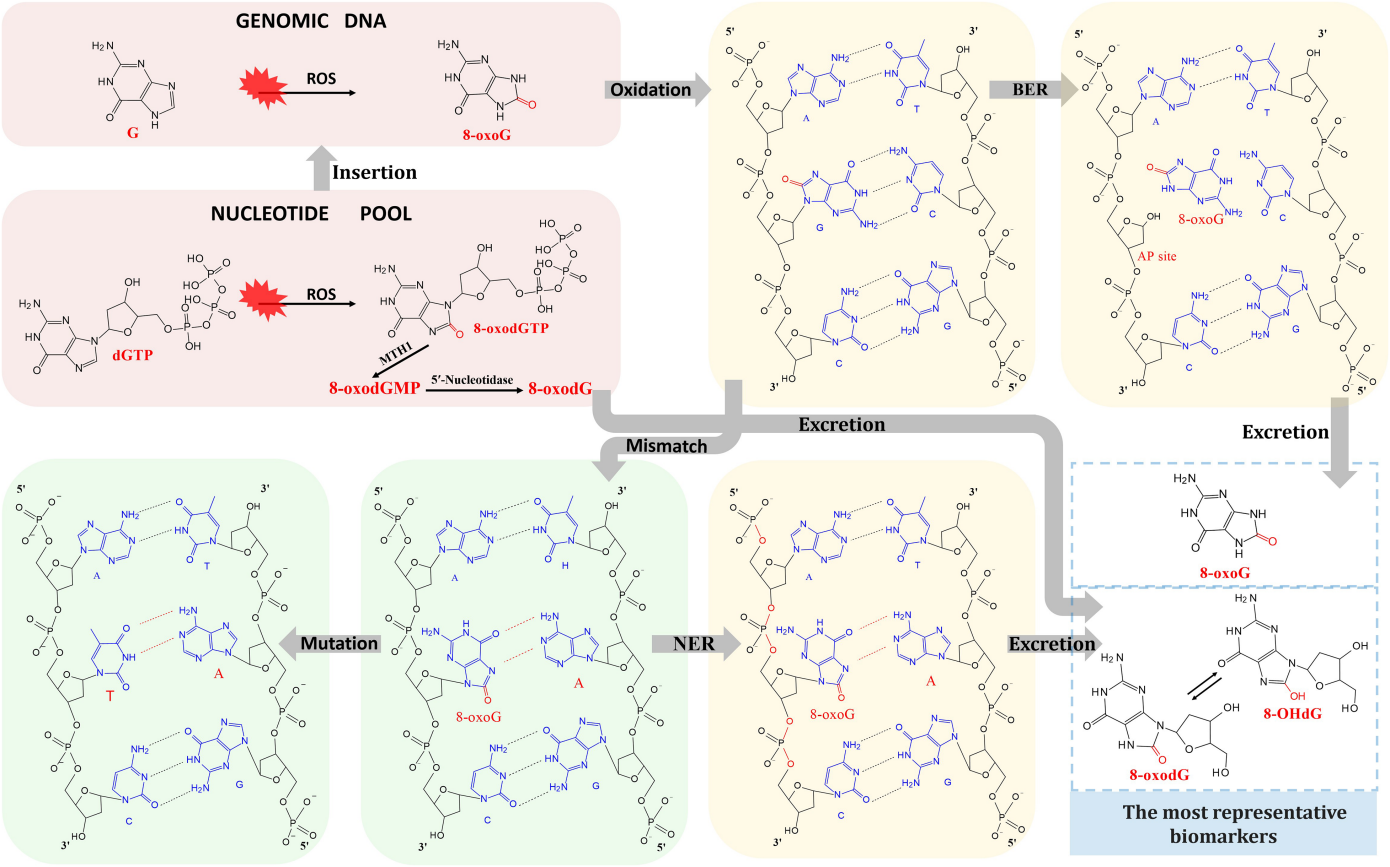
Mechanisms of DNA oxidation and the formation of 8-oxodG and 8-oHdG.

In the free nucleotide pool, guanine is oxidized to 8-oxo-dGTP and involved in DNA; in the DNA molecule, guanine is oxidized to 8-oxo-7,8-dihydroguanine (8-oxoG). The 8-oxo-dGTP in the nucleotide pool is first hydrolyzed to 8-oxodGMP by MutT homolog 1 (MTH1), followed by 8-oxo-2′-deoxyguanosine (8-oxodG or 8-OHdG) formation by 5′-Nucleotidase (237, 238). The base excision repair (BER) system recognizes and eliminates 8-oxoG from DNA strands. When the BER system fails to recognize larger or more complex damages, nucleotide excision repair (NER) is initiated to excise 8-oxodG-containing DNA fragments (237). In addition, cells with the most severe DNA damage (necrosis or apoptosis) will also release 8-oxodG-containing DNA fragments. The excision of 8-oxoG by the BER system also generates apurinic (AP) sites, which are highly reactive and susceptible to 3′ phosphate bond breaks, resulting in single-strand breaks. In the presence of oxygen, 8-oxoG readily produces a synconformation and pairs with adenine (226). Although DNA repair enzymes continuously monitor and repair chromosomes, 8-oxoG readily accumulates and induces deleterious mutations through free radical overload, causing guanine to thymine mutations and cytosine to adenine transversions during DNA replication, as shown in Figure 3. Thus, the major types of DNA damage following oxidation by ROS are oxidative base modifications (8-oxoG), AP sites, single-strand breaks, and mutations (G: C-T: A) (226). Whereas 8-oxoG, 8-oxodG, and 8-oxodG-containing DNA fragments are released into the bloodstream and are taken up by the kidneys and excreted into the urine, 8-oxoG and 8-oxodG are the most critical types of DNA damage, which have been extensively studied in tissues and body fluids (239).

## 5 Review of oxidative stress-related biomarkers for early prediction of BPD

We thoroughly searched the PubMed database for published clinical studies encompassing the neonatal (birth-1 month) up to 2023. Our search strategy incorporated medical subject headings (MeSH headings) and free-text terms associated with “BPD”, “lipid peroxidation”, “protein oxidative damage”, “nucleic acid oxidative damage”, and representative biomarkers. Our analysis was centered on identifying biomarkers applicable in clinical settings to forecast the progression of BPD. In total, we reviewed and summarized 37 clinical studies, which are detailed in Supplementary Table S1.

**Figure 4:**
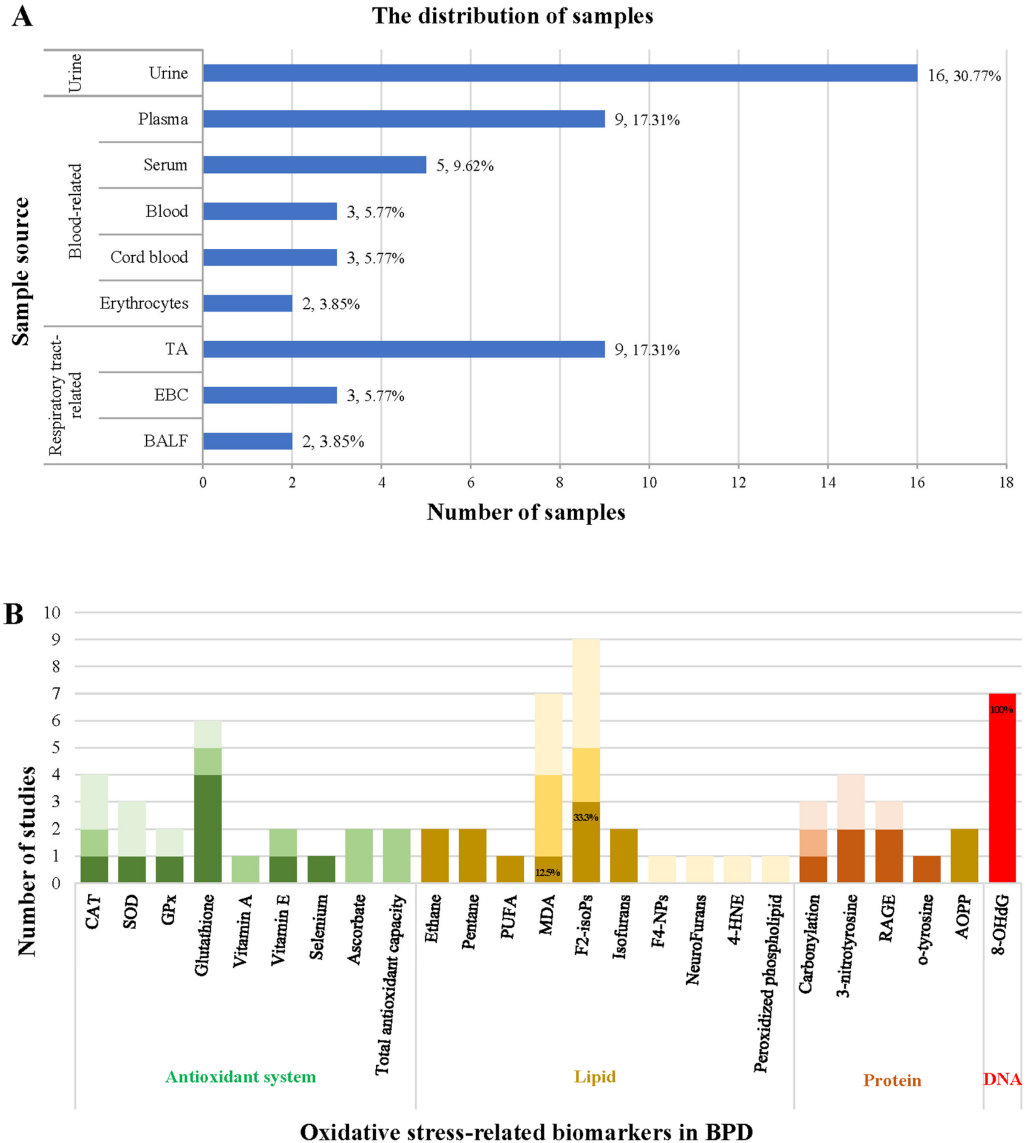
Summary of the source of samples **(A)** and the correlation of oxidative stress-related biomarkers with BPD **(B)**.

The publication dates of the articles ranged from 1988 to 2023 and involved 15 countries. Predominantly, studies were conducted in the USA (*n* = 11) and Finland (*n* = 4). The study subjects included premature and full-term infants, with sample sizes ranging from 19 to 253. The most common sample types used in the research were urine-derived (30.77%), blood-derived (42.31%), and respiratory-derived (26.92%) samples, including BALF, exhaled breath condensate (EBC), tracheal aspirates (TA), erythrocytes, cord, serum, blood, plasma, and urine. Among these, urine (30.77%), plasma (17.31%), and TA (17.31%) were the most frequently studied sample types, as shown in Figure 4A.

Our analysis reveals that biomarkers of lipid peroxidation were the most investigated assays, followed by antioxidant systems, protein oxidation, and nucleic acid oxidation. Notably, F2-isoPs (*n* = 9), MDA (*n* = 7), and 8-OHdG (*n* = 7) emerged as the most studied biomarkers, as illustrated in Figure 4B. We also elucidated the correlations between the antioxidant system, lipid peroxidation, protein oxidative damage, nucleic acid oxidative damage, and the development of BPD in Supplementary Table S1. Additionally, the accuracy of biomarkers associated with the antioxidant system, lipid peroxidation, and oxidative damage to proteins is depicted in Figure 4B. However, despite F2-isoPs and MDA being the two most extensively studied biomarkers of oxidative stress in these articles, only 33.3% and 12.5% of articles reported their predictive capability for BPD, respectively.

The darkest color blocks in Figure 4B indicate that the biomarkers can predict the development of BPD; darker color blocks indicate that the biomarkers show differences between premature infants and control groups or between different treatment groups but cannot predict the development of BPD; the lightest color blocks indicate that the biomarkers cannot predict the development of BPD.

Among the nine studies investigating F2-isoPs levels and their association with BPD, only two suggested that elevated plasma F2-isoPs levels can predict adverse outcomes in preterm infants (240, 241). One study indicated a correlation between increased F2-isoPs levels and a more minor gestational age (36). However, the remaining six reports concluded that F2-isoPs levels cannot predict the development of BPD in preterm infants (40, 242–246). Notably, two studies also examined the levels of other lipid peroxidation products, such as isofurans. They indicated that isofurans may exhibit more robust predictive capabilities, potentially emerging as the optimal biomarker for lipid peroxidation (40, 245). Considering the mechanism of lipid peroxidation, IsoFs may be more suitable than F2-isoPs as a lipid peroxidation metabolite for predicting BPD due to their specificity as a biomarker generated in the presence of increased oxygen concentration. Nonetheless, a limited number of studies investigating isofuran levels and their correlation with BPD emphasize the need for further research in this area (40, 245).

Seven studies have assessed MDA levels as a secondary product of lipid peroxidation. Among them, three reported that MDA levels could not predict BPD (135, 247, 248) and the other three showed that higher MDA concentrations are associated with gestational age (33, 35) or lower body weights (32). Only Weinberger et al. reported a correlation between elevated urinary MDA measurements and the risk for oxidative respiratory distress (249).

In contrast, elevated levels of 8-OHdG have been consistently associated with the clinical outcome of BPD across studies, suggesting that 8-OHdG could serve as a reliable biomarker for predicting BPD (37–40, 245, 250–252). Joung et al. demonstrated that the 8-OHdG values in “classic” BPD on the third day were higher than those of “atypical” BPD. Furthermore, 8-OHdG levels on the seventh day were an independent risk factor for developing moderate/severe BPD (250). Hsiao et al. reported higher 8-OHdG levels in serum and tracheal aspirates (TA) in the BPD group on the 1st day after birth (*p* < 0.05) and persistently 8-OHdG levels increased in TA fluid on the 28th day of life in the BPD group (*p* < 0.05) compared to the non-BPD group (37). Moreover, Hsiao et al. also suggested that urine 8-OHdG concentrations from days 14 to 28 may be practical non-invasive predictors of BPD development in preterm infants (39).

In a prospective cohort study, Vento et al. found that extremely preterm neonates receiving antenatal steroids had decreased 8-OHdG levels accompanied by increased antioxidant enzyme activity, lower ortho-tyrosine levels, and a lower incidence of BPD compared to those not receiving steroids (252). Tokuriki et al. demonstrated that urinary levels of 8-OHdG during the early postnatal period correlated with the subsequent development of BPD. In contrast, urinary levels of advanced oxidative protein products (AOPP) and N*ε*-(hexanoyl) lysine showed no such correlation (251), suggesting that 8-OHdG may be preferable to protein oxidation products as a predictor of oxidative stress biomarkers for BPD.

DNA oxidative stress biomarkers may offer greater accuracy in predicting the development of BPD for several reasons. (i) Location: Lipids and proteins in the cytoplasm or cell membrane are usually more susceptible to OS, significantly exacerbated by oxygen therapy (83, 201). In contrast, DNA, located in the nucleus and protected by the nuclear membrane, is less vulnerable to the effects of ROS. (ii) Biochemical properties: Lipids and proteins contain a large number of easily oxidizable groups, such as unsaturated fatty acids (201, 207, 253) and amino acids (207, 220), making them more prone to oxidation under hyperoxia. (iii) Metabolic activity: Lipids and proteins participate in metabolic processes that generate ROS and other oxidative substances, leading to oxidative damage. In contrast, DNA does not directly engage in metabolic reactions, reducing its exposure to oxidation (201). (iv) Biological importance: DNA integrity is crucial for proper cellular function and overall health, as it stores and transmits genetic information. Cells have evolved defense mechanisms, including enzymes and antioxidant systems, to repair and protect DNA from oxidative damage, whereas lipids and proteins lack comparable protective mechanisms (254). (v) Reflecting oxidative damage directly: DNA molecules have a more straightforward structure than lipids and proteins, making them more directly reflective of oxidative damage (201). (vi) Wide range of applications: 8-OHdG emerges as a pivotal biomarker with promising applications in disease research, clinical diagnostics, and environmental health assessments (255–257). However, its lack of tissue and diagnostic specificity poses challenges, necessitating consideration of other relevant factors when interpreting experimental results to accurately assess the extent of oxidative damage (243).

Based on our comprehensive review of clinical studies, it is evident that the relationship between many reported biomarkers and the outcome of BPD is ambiguous. Only 8-OHdG shows the most promise as an oxidative damage biomarker for predicting BPD and complications related to prematurity, and the relevant information is described in Table 1. However, its integration into clinical practice has been hindered by several factors, including the need for standardized methods and reference ranges and the absence of validation through prospective trials (258). Selecting sample sources and assays is crucial in obtaining convenient and reliable biomarkers for early BPD prognostic prediction in clinical settings. Five of the seven studies on 8-OHdG utilized urine samples (39, 40, 250–252), while the remaining two collected serum and tracheal aspirates (37, 38). The collection and processing of urine samples are relatively straightforward, usually involving minimal pre-processing steps. In contrast, collecting blood and BALF may compromise the integrity of the skin and mucosa, thereby raising the risk of pain and infection in premature infants (39). Consequently, urine samples are convenient for large-scale research and clinical monitoring purposes. Urinary 8-OHdG is generally regarded as a reflection of systemic oxidative stress, thereby serving as an indicator for assessing overall oxidative stress status (256). Rigorous analytical methods and quality control measures are imperative to ensure a reliable assessment of 8-OHdG levels, providing researchers and clinicians with a basis for optimizing neonatal care. Five of the seven studies investigating 8-OHdG utilized ELISA assay kits for measurement (37–39, 250, 251), while the remaining two employed mass spectrometry (40, 252). Although ELISA assays have been calibrated for measurements and proven helpful in assessing the impact on BPD development and progression, there remains a necessity for more sensitive, specific, and clinically validated laboratory detection methods. Techniques like HPLC-MS and LC-MS/MS hold promise in obtaining urinary 8-OHdG biomarkers from urine samples, thereby enhancing the accuracy and reliability of BPD prediction (256, 259).

**Table 1:** Summary of studies reporting on 8-oHdG in BPD.

Country of publication	Publication date	Purpose of study	Subjects	Sample type	Sample collection time	Test indicators	8-OHdG concentrations	Cite
Korea	2011	Compare urinary inflammatory and oxidative stress markers between BPD groups	60 Preterm infants <30 weeks gestation or <1,250 g (24 “atypical” BPD and 36 ‘classic’ BPD)	Urine	Days 1, 3 and 7 of life	Enzyme-linked immunosorbent assay (JalCA, Fukuroi, Shizuoka, Japan)	No/mild BPD group (1.6 ng/mg) vs. moderate/severe group (2.8 ng/mg) on Day 7 after birth, *p* = 0.002.	(250)
Japan	2015	To evaluate carboxyhemoglobin (CO-Hb) levels as a biomarker for predicting BPD development and severity	25 Preterm infants <33 weeks gestation and/or <1,500 g (16 No-or-mild BPD and 9 Moderate-to-severe BPD)	Urine	Postnatal days 5–8 and 26–29	Enzyme-linked immunosorbent assay (JalCA, Fukuroi, Shizuoka, Japan)	The moderate-to-severe BPD group [18.8 (13.1–86.6) ng/mg] vs. the no-or-mild BPD group [11.9 (3.6–26.6) ng/mg] on Day 5–8 after birth, *p* < 0.05.	(251)
China	2017	Compare changes between IL-6 and oxidative stress marker with 8-OHdG in VLBW preterm infants following development of BPD.	80 VLBW preterm infants (26 BPD and 54 Non-BPD)	Serum	Day 1 and Day 28 after birth	Enzyme-linked immunosorbent assay (JalCA, Fukuroi, Shizuoka, Japan)	The moderate-to-severe BPD group [19.6 (9.8–176.8) ng/ml] vs. the non-BPD group [18.8 (5.9–50.6) ng/ml] on Day 1 after birth, *p* < 0.05; the moderate-to-severe BPD group [39.5 (11.3–115.4) ng/ml] vs. the non-BPD group [17.3 (3.8–51.6) ng/ml] on Day 28 after birth, *p* < 0.05.	(37)
China	2021	Examine Hsp-70 and 8-OHdG from TA in VLBW preterm infants to predict BPD	109 VLBW preterm infants (32 BPD and 77 Non-BPD)	TA	Day 1 and Day 28	Enzyme-linked immunosorbent assay (JalCA, Fukuroi, Shizuoka, Japan)	The BPD group (20.9 ± 8.9 ng/mg) vs. the non-BPD group (14.8 ± 10.4 ng/mg) on Day 1 after birth, *p* < 0.05; the BPD group (42.0 ± 28.5 ng/mg) vs. the non-BPD group (14.1 ± 10.6 ng/mg) on Day 28 after birth, *p* < 0.05.	(38)
China	2022	Predict BPD in preterm infants using urinary 8-OHdG and NT-proBNP	165 Preterm infants <33 weeks gestation or <1,500 g (70 BPD and 95 Non-BPD)	Urine	Days 7, 14, 21 and 28 after birth	Enzyme-linked immunosorbent assay (Uscn Life Science Inc., Wuhan, P.R. China)	The BPD group (19.34 ± 2.24 ng/mg) vs. the non-BPD group (17.63 ± 1.59 ng/mg) on Day 7 after birth, *p* < 0.05; the BPD group (26.48 ± 4.92 ng/mg) vs. the non-BPD group (20.24 ± 2.93 ng/mg) on Day 14 after birth, *p* < 0.05; the BPD group (27.55 ± 3.66 ng/mg) vs. the non-BPD group (20.86 ± 3.28 ng/mg) on Day 21 after birth, *p* < 0.05; the BPD group (23.95 ± 4.06 ng/mg) vs. the non-BPD group (17.21 ± 2.75 ng/mg) on Day 28 after birth, *p* < 0.05.	(39)
Spain	2009	To study the association between antenatal steroids and antioxidant activity, and their impact on postnatal oxidative stress	57 Preterm infants <28 weeks gestation (37 receiving antenatal steroids and 20 not receiving antenatal steroids)	Urine	At birth	Mass spectrometry (8OHdG/1dG ratio)	Group receiving antenatal steroids (6.73 ± 2.18) vs. group not receiving antenatal steroids (9.53 ± 3.83) at birth, *p* < 0.01	(252)
Spain	2009	Reduce adverse pulmonary outcomes, oxidative stress, and inflammation in infants of 24–28 weeks of gestation	78 Preterm infants of 24–28 weeks gestation (37 infants receiving 30% oxygen and 41 infants receiving 90% oxygen)	Urine	Days 1 and 7 of life	Mass spectrometry (8OHdG/2dG ratio)	Group receiving 30% oxygen (about 12.5) vs. group receiving 90% oxygen (about 19) on Day 1 after birth, *p* < 0.01; group receiving 30% oxygen (about 24) vs. group receiving 90% oxygen (about 32) on Day 7 after birth, *p* < 0.01.	(40)

Since the levels of oxidative stress-induced biomarkers can reflect the severity of BPD, their levels can guide clinical management decisions for premature infants, thereby reducing oxidative stress-related diseases and providing valuable insights into the treatment of oxidative stress-related neonatal diseases (260). Currently, various strategies have been proposed for treating neonatal BPD, including protective ventilation, surfactant therapy, corticosteroids, caffeine, vitamin A, nitric oxide, and nutritional interventions (261). Therefore, assessing the relationship between these strategies and oxidative stress levels or BPD incidence is critical. Ten studies have described the changes in oxidative stress biomarkers following drug administration (252, 262), inhaled nitric oxide therapy (34, 263), exposure to different oxygen concentrations (40, 245, 246), and nutritional interventions (243, 248, 264), and their relationship with the outcome of BPD.

Reports suggest that using corticosteroids improves the oxidative-reductive balance, resulting in increased antioxidant enzyme activity, decreased 8-OHdG levels, lower ortho-tyrosine, and a reduced incidence of BPD (252). Although no significant differences in clinical outcomes were observed between control and beclomethasone-treated infants, bronchoalveolar lining fluid analysis revealed evidence of phospholipid peroxidation in control infants compared to beclomethasone-treated infants on day 2 of life (262).

There were no significant differences in concentrations of 3-nitrotyrosine and carbonylation between control and inhaled nitric oxide-treated infants (34, 263).

A study conducted in Spain in 2009 showed that urinary markers of oxidative stress were significantly elevated in infants receiving 90% oxygen compared to those receiving 30% oxygen in the first week after birth. Additionally, GSSG levels on day three and urinary isofuran, o-tyrosine, and 8-oxodG levels on day seven significantly correlated with chronic lung disease development. However, the groups had no differences in urinary F2-isoPs levels (40). Furthermore, another study from Spain in 2015 reported no differences in oxidative stress biomarkers, mortality, or major perinatal morbidities between infants receiving 30% oxygen and those receiving 60%–65% oxygen. Nonetheless, isofurans detected in the first 4 days after birth were correlated with the later development of BPD compared to F2-isoPs, F4-NPs, and NeuroFurans (245). However, a recent study from India indicated that in premature infants receiving room air vs. 100% oxygen therapy within 4 h after birth, F2-isoPs levels showed no significant difference in mortality or BPD (246).

Unfortunately, nutritional supplementation does not reduce the incidence of BPD significantly. A study from Canada in 2010 suggested that while the LIP + MVP TPN modality may help protect against the oxidant load associated with oxygen supplementation, its effectiveness in reducing the incidence of BPD remains unclear (243). Another study from Turkey in 2019 demonstrated that total antioxidant capacity was higher in the SMOFlipid group compared with the ClinOleic group on day 7. Although BPD was lower in the SMOFlipid group than in the ClinOleic group, this finding was non-significant (264). Similarly, another study from Turkey in 2019 indicated that FMOS and OO/SO lipid emulsions had similar effects on lipid peroxidation on the 28th day of life and short-term morbidities such as BPD (248).

## 6 Conclusions

We have been grappling with BPD since Northway first outlined it in 1967, spanning over 60 years. BPD stands out as the most prevalent chronic lung disease affecting premature infants, with a solid correlation to significant morbidity and mortality. The pathogenesis of BPD is complex, primarily characterized by increased exposure to oxidative stress and immature antioxidant systems, ultimately resulting in abnormal pulmonary and vascular growth. Oxidative stress triggers oxidative damage to lipids, proteins, and nucleic acids. Numerous studies have shown elevated levels of oxidative stress markers in newborns who develop BPD. Thus, monitoring these markers in premature infants helps predict the severity of BPD and evaluate the effectiveness of therapeutic interventions.

With advancements in medical care, various approaches, including pulmonary surfactants, vitamin A, vitamin E, vitamin D, caffeine, and nutritional interventions, have been utilized in the clinical management of BPD. However, the efficacy and safety of some of these methods remain contentious and necessitate further evaluation. Considering the pivotal role of oxidative stress in BPD pathogenesis, interpreting clinical treatment strategies from an antioxidant perspective is justifiable.

Furthermore, we analyzed 37 clinical studies on oxidative stress markers to investigate the relationship between lipid peroxidation, protein oxidation, oxidative DNA damage, and the outcome of BPD in newborns. 8-OHdG is the most informative among these biomarkers, elucidating the disease severity and enabling personalized precision treatment for affected infants.

Overall, our review contributes to a deeper understanding of BPD pathogenesis stemming from continuous hyperoxia exposure during treatment. It sheds light on the mechanisms underlying the antioxidant aspects of clinical BPD management. As pathogenesis is not fully understood, it is promising to explore new evidence related to oxidative stress and the pathogenesis of BPD from the perspective of microbiomics, metabolomics, and proteomics (265).
