# Supplementary material for: Research progress on pathophysiologic mechanisms, clinical treatment and predictive biomarkers in bronchopulmonary dysplasia: from the perspective of oxidative stress
Source: Front Pediatr. 2025 Mar 27;12:1343870. doi: 10.3389/fped.2024.1343870 (PMC11981909; doi:10.3389/fped.2024.1343870)
Supplement: Supplementary file 1 [file Table1.pdf]

Supplementary Material

1 Supplementary Tables

Supplementary Tables 1: Summary of studies reporting on oxidative stress biomarkers in BPD

**Country of Publication**	**Publication Date**	**Purpose of Study**	**Subjects**	**Sample Type**	**Sample Collection Time**	**Test Indicators**	**Indicators with Significant Differences**	**Cite**
USA	1988	Examine the effects of inhaled beclomethasone (BEC) on bronchoalveolar lining fluid oxyradical inflammation in premature infants at risk for BPD.	18 pemature infants at risk for bronchopulmonary dysplasia (9 BEC and 9 Control)	TA	Days 2, 4, and 6 of life	lnterleukin-8, Myeloperoxidase, Peroxidized phospholipid	Although no differences in clinical outcome were apparent in comparing nine control infants with nine beclomethasone-treated infants, bronchoalveolar lining fluid from control infants exhibited evidence of apparent phospholipid peroxidation (enhanced polyunsaturated fatty acid consumption) on Day 2 of life compared to beclomethasone-treated infants.	[1]

Finland	1990	Measure lipid peroxidation in very low birth weight infants and correlate it with clinical outcomes.	19 Very low birth weight infants (11 BPD and 8 Non-BPD)	Exhaled breath condensate	First 5 days postnatally	Ethane, Pentane	Expired ethane and pentane were significantly higher for patients with a poor outcome (deaths or bronchopulmonary dysplasia) than for those with good outcomes.	[2]
Finland	1994	Examine the role of immaturity in free radical-mediated lipid peroxidation in premature infants.	27 Preterm infants <32 weeks gestational age (18 Non-BPD, 9 BPD)	Exhaled breath condensate	First 18 days of life	Ethane, Pentane	Maximum ethane and pentane correlated with gestational age and birth weight. Infants with high maximum expired ethane and pentane had higher odds of dying or having bronchopulmonary dysplasia.	[3]
Finland	1995	Study pulmonary protein oxidation & its association with development of chronic lung disease in infants receiving oxygen therapy	61 Newborn infants requiring intensive care with oxygen therapy (24 to 41 weeks' gestation)	TA	During the first week of life	**Carbonylation**	Oxidation of proteins is related to the development of chronic lung disease.	[4]
USA	1998	Investigate the role of peroxynitrite in the etiology of BPD by measuring levels of plasma 3-nitrotyrosine	26 Preterm infants < 30 weeks' gestation (10 BPD and 16 Non-BPD)	Plasma	First month of life	**3-nitrotyrosine**	3-nitrotyrosine may be useful as an early plasma indicator of infants at risk for developing BPD.	[5]

Germany	2000	Investigate whether low concentrations of polyunsaturated fatty acids (PUFA) and dimethylacetals (DMA, representing plasmalogens) in TA both were associated with development of BPD and whether both were further reduced during mechanical ventilation with oxygen in extremely preterm infants.	25 Preterm infants <32 weeks gestational age (15 Non-BPD and 10 BPD)	TA	Immediately after birth and in the following 4 days	PUFA, DMA	Lower PUFA and DMA percentages in infants who developed BPD compared to non-BPD infants.	[6]
New Zealand	2000	Determine if oxidative injury markers (protein carbonyls and MDA) are elevated in TA from very low birth weight infants, and if they correlate with myeloperoxidase and poor respiratory outcomes.	140 infants (86 infants < 1500 g and 54 infants > or = 1500 g)	TA	soon after birth	**Carbonylation**, **MDA**, Myeloperoxidase	Protein carbonyls and MDA were significantly higher in infants < 1500 g than larger infants, and were highest close to birth. Oxidative marker levels were not significantly associated with the development of chronic lung disease.	[7]
USA	2003	Study pulmonary plasma levels of 3-nitrotyrosine and its association BPD	16 Preterm infants of 24-32 weeks' gestation (10 BPD and 6 Non-BPD)	Plasma	0 and 72 h after initiation of inhaled nitric oxide	**3-nitrotyrosine**, 3-chlorotyrosine	Infants whose 3-nitrotyrosine levels decreased over the 72 h period were more likely to wean off of mechanical	[8]

							ventilation (p = 0.03).	
USA	2003	To determine if premature infants who developed BPD demonstrate lower levels of antioxidants and lower level of lipid peroxidation	81 Premature infants (23 RDS, 23 BPD and 35 healthy controls)	Cord, plasma, erythrocytes and urine	First 3 days of life	Blood levels of Selenium, Vitamin E, Glutathione, SOD, **urine levels of MDA**	Low plasma selenium and Vitamin E levels were significantly associated with increased respiratory morbidity. There was no relationship between lipid peroxidation and BPD.	[9]
USA	2004	Analyze the correlation of early urinary MDA and TBARS with prenatal betamethasone administration and with the development of 'oxygen radical diseases' (ORD).	25 Preterm infants <30 weeks gestation	Urine	During the first 10 days of life	**MDA**	Elevated urinary MDA measurements in the first 10 days are correlated with the risk for ORD.	[10]
UK	2004	Determine the relation between lipid peroxidation and antioxidants in ventilated premature infants and relate it to clinical outcome.	43 ventilated infants <32 weeks gestation (17 control, 8 Non-BPD, 18 BPD and 5 dead)	BALF	Weekly from birth	Ascorbate, Glutathione, Urate, **MDA**	Though infants who developed BPD had significantly lower initial Glutathione and higher MDA concentrations in the epithelial lining fluid than those who were not oxygen	[11]

							dependent, these variables were poor predictors of the development of BPD.	
Finland	2004	Whether the plasma free F2-isoP and ascorbate radicals can predict oxidative damage in very low birth weight infants and the effect of N-acetylcysteine (NAC) on these indices.	83 ELBW infants (54 Non-BPD and 29 BPD or died)	Plasma	Days 3 and 7 of life	**F2-isoPs**, Ascorbate	Plasma free F2-isoPs could serve as a marker in assessing the risk for BPD. NAC treatment or the later development of BPD was not related to the ascorbyl radical levels.	[12]
USA	2007	Determine if urinary F2-IsoP levels are higher in infants who develop BPD	40 Preterm infants <30 weeks gestation (24 BPD and 16 Non-BPD)	Urine	Weekly from birth	**F2-isoPs**	Urinary F2-isoPs in early postnatal life in preterm infants is not correlated with the development of BPD.	[13]
USA	2008	Examine effects of inhaled nitric oxide therapy on oxidative stress in premature infants	100 Preterm infants of 22.7-30 weeks' gestation (49 in Control group and 51 in iNO trrated group)	Blood	Days 1, 7 and 10 of life	**3-nitrotyrosine, Carbonylation**	Plasma carbonyl was positively correlated with severity score. Plasma 3-nitrotyrosine was not significantly correlated with severity score. No significant differences between control and iNO-treated infants for concentrations of 3-nitrotyrosine and carbonylation.	[14]

Spain	2009	To study the association between antenatal steroids and antioxidant activity, and their impact on postnatal oxidative stress	57 Preterm infants < 28 weeks gestation (37 receiving antenatal steroids and 20 not receiving antenatal steroids)	Cord blood	At birth	SOD, CAT, GPX, Glutathione, **ortho-tyrosine**, **8-OHdG**	The usage of antenatal steroids are accompanied by increased antioxidant enzyme activity, decreased 8-OHdG levels, lower ortho-tyrosin and lower incidence of BPD.	[15]
Spain	2009	Reduce adverse pulmonary outcomes, oxidative stress, and inflammation in infants of 24 to 28 weeks of gestation	78 Preterm infants of 24 to 28 weeks gestation (37 infants receiving 30% oxygen and 41 infants receiving 90% oxygen)	Blood, urine and plasma	Days 1 and 7 of life	Blood oxidized Glutathione (GSSG)/reduced Glutathione ratio, **urinary ortho-Tyrosine**, **urinary 8-oxodG**, **urinary F2-isoPs**, **Isofuran**, plasma interleukin 8 and tumor necrosis factor alpha levels	Urinary markers of oxidative stress were increased significantly in the high-oxygen group compared with the low-oxygen group in the first week after birth. GSSG levels on day 3 and urinary isofuran, o-tyrosine, and 8-oxodG levels on day 7 were correlated significantly with development of chronic lung disease. There was no differences in urinary isoprostane metabolite levels between the groups.	[16]
Italy	2010	To test the hypothesis that oxidative stress (OS) markers in cord blood can predict the	168 Preterm infants of 24-32 weeks' gestation (135	Cord blood	At birth	non-protein bound iron, basal superoxide anion, stimulation superoxide anion, total	The development of FRD was significantly associated to high	[17]

		onset of free radical-related diseases (FRD)	Normal infants and 33 Affected infants)			hydroperoxides, **AOPP**	cord blood levels of total hydroperoxides, AOPP and non-protein bound iron.	
Canada	2010	To compare markers of oxidant stress in premature infants receiving lipid emulsion (LIP)+parenteral multivitamins (MVP), either exposed to or protected from light, or amino acid solution (AA)+MVP.	56 Preterm infants < 27 weeks' gestation (16 in AA + MVP group, 17 in LIP + MVP + ambient light, and 19 in LIP + MVP + Photoprotection)	Blood and urine	Days 7 and 10 of life	Vitamin A, Vitamin E, the redox potential of Glutathione, **F2-isoPs, Dityrosine**	There is a strong association between **elevated redox potential** and late BPD. Isoprostane and dityrosine levels were not associated with severity of later BPD.	[18]
Korea	2011	Compare urinary inflammatory and oxidative stress markers between BPD groups	60 Preterm infants <30 weeks gestation or < 1250 g (24 'atypical' BPD and 36 'classic' BPD)	Urine	Days 1, 3 and 7 of life	Leukotriene E4, **8-OHdG**	In 'classic' BPD, the 8-OHdG values on the 3(rd) day were higher than those of 'atypical' BPD. The 8-OHdG levels on the 7 day were the independent risk factor for developing moderate/severe BPD. These results suggest that oxidative DNA damage could be the crucial mechanism in the pathogenesis of current BPD.	[19]

USA	2012	Measure F2-isoP in children with IPH and BPD	37 infants (5 IPH, 12 BPD with PH and 20 control)	Plasma	Not specified	**F2-isoPs**	IPH group had higher F2-isoPs than controls; BPD group with PH had lower F2-isoPs than controls.	[20]
Italy	2015	Investigate associations between BPD and lipid hydro-peroxide (LOOH) and glutathione (GSH) concentrations in BALF	44 preterm infants with RDS and (11 BPD and 33 controls)	BALF	Within two hours of birth	LOOH, Glutathione	BPD could be predicted early by evaluating early LOOH level that increases in preterm infants developing BPD.	[21]
Japan	2015	To evaluate carboxyhemoglobin (CO-Hb) levels as a biomarker for predicting bronchopulmonary dysplasia (BPD) development and severity	25 Preterm infants < 33 weeks gestation and/or < 1500 g (16 No-or-mild BPD and 9 Moderate-to-severe BPD)	Blood, Urine	Postnatal days 5-8, 12-15, 19-22, 26-29	CO-Hb, **8-OHdG**, **AOPP, Nε-(hexanoyl) lysine**	CO-Hb levels during the early postnatal period may serve as a practical marker for evaluating oxidative stress and the severity of subsequently developing BPD. Urinary levels of 8-OHdG during the early postnatal period correlated with the subsequent development of BPD, but urinary levels of advanced oxidative protein products (AOPP) and Nε-(hexanoyl) lysine	[22]

							(HEL) showed no such correlation.	
Spain	2015	Evaluate in vivo oxidative stress in preterm infants	253 Preterm infants <32 weeks gestation (133 infants receiving 30% oxygen and 120 infants receiving 60-65% oxygen)	Urine	during the first 4 weeks after birth	**F2-isoPs, Isofurans, F4-NPs, NeuroFurans**	Tough no significant differences in oxidative stress biomarkers, mortality, or major perinatal morbidities were found, urinary **elimination of isofurans** in the first 4 days after birth correlated with later development of bronchopulmonary dysplasia.	[23]
USA	2016	Examine the association between early oxidative stress and later morbidity	136 Preterm infants <28 weeks gestation	Plasma	Days 14 and 28 after birth	**F2-isoPs**	Increase in F2-isoPs associated with decreased developmental scores and increased respiratory morbidity.	[24]
Egypt	2016	Assess levels of antioxidants and a marker of lipid peroxidation in preterm infants	200 infants (100 preterm infants and 100 full-term infants)	Plasma	At birth	Vitamin A, Vitamin E, CAT, Total antioxidant capacity, **MDA**	The plasma levels of vitamin A, vitamin E, TAS and catalase were significantly lower in the preterm than in the full-term group, and the plasma level of MDA was significantly higher in preterm	[25]

							than full-term infants.	
China	2017	Compare changes between IL-6 and oxidative stress marker with 8-OHdG in VLBW preterm infants following development of BPD.	80 VLBW preterm infants (26 BPD and 54 Non-BPD)	Serum and TA	Day 1 and Day 28 after birth	**8-OHdG**	IL-6 and 8-OHdG in serum and TA were higher in the BPD group than in the non-BPD group on the 1st day after birth ($p < 0.05$). The IL-6 and 8-OHdG levels in TA fluid were persistently increased on the 28th day of life in the BPD group ($p < 0.05$).	[26]
USA	2017	Investigate the presence of sRAGE in ventilated infants	19 Preterm infants (7 Non-BPD, 10 BPD and 5 dead)	TA	First week or fifth week of life	**sRAGE**	The sRAGE band densities were similar among the seven infants who fully recovered, eight who developed bronchopulmonary dysplasia (BPD), and 5 who died (analysis of variance p = 0.797).	[27]
Germany	2017	Determine the effects of iNO on pulmonary inflammation	43 Preterm infants of 24-28 weeks' gestation (18 in Control group and 25 in iNO trrated group)	TA	Postnatal day 2 to 14	IL-1β, IL-6, IL-8, TGF-β1, IP-10, MIP-1α, ASM, Neuropeptide Y, Leukotriene B4, **Nitrotyrosine**, Nitrite	Nitrotyrosine TA concentrations and incidence of BPD were not affected by iNO treatment.	[28]

USA	2018	Quantify sRAGE levels in the lungs of intubated preterm infants and examine its association with severe BPD.	45 Preterm infants born (26 control and 19 BPD)	TA	1 week of age	**esRAGE, sRAGE**	A twofold increase in esRAGE or total sRAGE was associated with decreased adjusted odds of severe BPD/death.	[29]
India	2018	To determine the incidence and associations of BPD in preterm infants	250 Preterm infants <33 weeks gestation (80 developed BPD and 170 Non-BPD)	Serum	Day 3 of life	**MDA**, CAT	Serum MDA, SOD and Catalase levels were comparable between the two groups.	[30]
Turkey	2019	Compare the effectiveness of a new-generation lipid emulsion (SMOFLipid) and olive-oil based lipid emulsion (ClinOleic) for preventing PN-associated oxidative damage in preterm infants.	89 Preterm infants < 32 weeks gestation (42 in SMOFlipid group and 47 in ClinOleic group)	Blood and urine	At 0 h, days 7 and 14	Total antioxidant capacity, anti- and pro-inflammatory cytokines (IL-6, IL-10, IL-1B, TNFα)	Total antioxidant capacity was higher in the SMOFlipid group compared with the ClinOleic group on day 7. BPD was lower in the SMOFlipid group (14.1%) than in the ClinOleic group (31.2%), but this finding was non-significant p > 0.05.	[31]
Turkey	2019	To investigate the effects of Olive oil-soybean oil (OO/SO) and Fish oil, MCT, Olive oil and Soya oil (FMOS) lipid preparations on cholesTAis, antioxidant enzymes	67 Preterm infants < 32 weeks gestation and/or < 1500 g (34 patients in FMOS groupand 33 patients in OO/SO lipid group)	Erythrocytes	First day of life, 7th day of lipid use, 28th day of life	CAT, SOD, Glutathione peroxidase, **MDA**	FMOS and OO/SO lipid emulsions have similar effects on lipid peroxidation on 28th day of life and on morbidities in short term period (BPD).	[32]

		levels, and lipid peroxidation						
Japan	2021	Investigate serum sRAGE levels at birth and its potential as a biomarker for BPD.	84 Preterm infants <32 weeks gestational age (50 Non-BPD, 34 BPD) and 40 healthy infants	Serum	At birth	**sRAGE**	Serum sRAGE levels at birth could serve as a biomarker for predicting BPD, but not its severity.	[33]
China	2021	Examine Hsp-70 and 8-OHdG from TA in VLBW preterm infants to predict BPD	109 VLBW preterm infants (32 BPD and 77 Non-BPD)	TA	Day 1 and Day 28	Hsp-70, **8-OHdG**	Multiple linear regression analysis demonstrated that BPD was significantly associated with gestational age, respiratory distress syndrome, and TA Hsp-70 and 8-OHdG levels on post-natal Day 28.	[34]
China	2022	Predict BPD in preterm infants using urinary 8-OHdG and NT-proBNP	165 Preterm infants <33 weeks gestation or <1500 g (70 BPD and 95 Non-BPD)	Urine	Days 7, 14, 21 and 28 after birth	**8-OHdG**, NT-proBNP	The urine 8-OHdG concentrations from 14 to 28 may be practical non-invasive predictors of BPD development in preterm infants.	[35]
Australia	2023	Assess if inflammatory markers were elevated in exhaled breath condensate (EBC) of infants born very	38 infants (15 Term-born infants, 11 Non-BPD and 12 BPD)	Exhaled breath condensate	12-16 corrected months of age	Leukotriene B4, **F2-isoPs**	Levels of Leukotriene B4 and F2-isoPs were elevated in preterm-born infants compared to term-	[36]

		prematurely (< 32 weeks gestation) at 12-16 corrected months of age and if increased levels were associated BPD diagnosis and respiratory morbidity.					born infants. But levels were independent of BPD diagnosis and respiratory morbidity during infancy.	
India	2023	Compare room air with 100% oxygen for oxidative stress and clinical outcomes in resuscitation of preterm infants	124 Preterm infants of 28-33 weeks' gestation (59 receiving room air and 65 receiving 100% oxygen)	Plasma	At 4 hours of age	**F2-isoPs**	No difference was observed in mortality by discharge and BPD.	[37]

Biomarkers of the antioxidant system, lipid peroxidation, protein oxidative damage, and DNA oxidative damage are color-coded in green, gold, orange, and purple, respectively. The representative biomarkers are in bold. Other biomarkers are marked in black. Outcomes detected in red font can predict the development of BPD, those in pink font indicate that the detected biomarkers show differences between premature infants and control groups or between different treatment groups but cannot predict the development of BPD, and those in blue font indicate that the detected biomarkers cannot predict the development of BPD.

[1] J.J. Zimmerman, D. Gabbert, C. Shivpuri, S. Kayata, W. Ciesielski, J. Miller, M.E. Peters, R.P. Eissenstat, and G. Shen, Meter-dosed, inhaled beclomethasone attenuates bronchoalveolar oxyradical inflammation in premature infants at risk for bronchopulmonary dysplasia. Am. J. Perinatol. 15 (1998) 567-76.

[2] O.M. Pitkanen, M. Hallman, and S.M. Andersson, Correlation of free oxygen radical-induced lipid peroxidation with outcome in very low birth weight infants. J. Pediatr. 116 (1990) 760-4.

[3] E. Varsila, O. Pitkanen, M. Hallman, and S. Andersson, Immaturity-dependent free radical activity in premature infants. Pediatr. Res. 36 (1994) 55-9.

[4] E. Varsila, E. Pesonen, and S. Andersson, Early protein oxidation in the neonatal lung is related to development of chronic lung disease. Acta Paediatr. 84 (1995) 1296-9.

[5] B.A. Banks, H. Ischiropoulos, M. McClelland, P.L. Ballard, and R.A. Ballard, Plasma 3-nitrotyrosine is elevated in premature infants who develop bronchopulmonary dysplasia. Pediatrics 101 (1998) 870-4.

[6] M. Rudiger, A. von Baehr, R. Haupt, R.R. Wauer, and B. Rustow, Preterm infants with high polyunsaturated fatty acid and plasmalogen content in tracheal aspirates develop bronchopulmonary dysplasia less often. Crit. Care Med. 28 (2000) 1572-7.

[7] I.H. Buss, B.A. Darlow, and C.C. Winterbourn, Elevated protein carbonyls and lipid peroxidation products correlating with myeloperoxidase in tracheal aspirates from premature infants. Pediatr. Res. 47 (2000) 640-5.

[8] S.A. Lorch, B.A. Banks, J. Christie, J.D. Merrill, J. Althaus, K. Schmidt, P.L. Ballard, H. Ischiropoulos, and R.A. Ballard, Plasma 3-nitrotyrosine and outcome in neonates with severe bronchopulmonary dysplasia after inhaled nitric oxide. Free Radic. Biol. Med. 34 (2003) 1146-52.

[9] H.S. Falciglia, J.R. Johnson, J. Sullivan, C.F. Hall, J.D. Miller, G.C. Riechmann, and G.A. Falciglia, Role of antioxidant nutrients and lipid peroxidation in premature infants with respiratory distress syndrome and bronchopulmonary dysplasia. Am. J. Perinatol. 20 (2003) 97-107.

[10] B. Weinberger, M. Anwar, S. Henien, A. Sosnovsky, M. Hiatt, N. Jochnowitz, G. Witz, and T. Hegyi, Association of lipid peroxidation with antenatal betamethasone and oxygen radial disorders in preterm infants. Biol Neonate 85 (2004) 121-7.

[11] K.J. Collard, S. Godeck, J.E. Holley, and M.W. Quinn, Pulmonary antioxidant concentrations and oxidative damage in ventilated premature babies. Arch. Dis. Child. Fetal Neonatal Ed. 89 (2004) F412-6.

[12] T. Ahola, V. Fellman, I. Kjellmer, K.O. Raivio, and R. Lapatto, Plasma 8-isoprostane is increased in preterm infants who develop bronchopulmonary dysplasia or periventricular leukomalacia. Pediatr. Res. 56 (2004) 88-93.

[13] S.D. Reuter, D.J. O'Donovan, S.E. Hegemier, E.O. Smith, W.C. Heird, and C.J. Fernandes, Urinary F2-isoprostanes are poor prognostic indicators for the development of bronchopulmonary dysplasia. J. Perinatol. 27 (2007) 303-6.

[14] P.L. Ballard, W.E. Truog, J.D. Merrill, A. Gow, M. Posencheg, S.G. Golombek, L.A. Parton, X. Luan, A. Cnaan, and R.A. Ballard, Plasma biomarkers of oxidative stress: relationship to lung disease and inhaled nitric oxide therapy in premature infants. Pediatrics 121 (2008) 555-61.

[15] M. Vento, M. Aguar, J. Escobar, A. Arduini, R. Escrig, M. Brugada, I. Izquierdo, M.A. Asensi, J. Sastre, P. Saenz, and A. Gimeno, Antenatal steroids and antioxidant enzyme activity in preterm infants: influence of gender and timing. Antioxid. Redox Signal. 11 (2009) 2945-55.

[16] M. Vento, M. Moro, R. Escrig, L. Arruza, G. Villar, I. Izquierdo, L.J. Roberts, 2nd, A. Arduini, J.J. Escobar, J. Sastre, and M.A. Asensi, Preterm resuscitation with low oxygen causes less oxidative stress, inflammation, and chronic lung disease. Pediatrics 124 (2009) e439-49.

[17] S. Perrone, M.L. Tataranno, S. Negro, M. Longini, B. Marzocchi, F. Proietti, F. Iacoponi, S. Capitani, and G. Buonocore, Early identification of the risk for free radical-related diseases in preterm newborns. Early Hum. Dev. 86 (2010) 241-4.

[18] P. Chessex, C. Watson, G.W. Kaczala, T. Rouleau, M.E. Lavoie, J. Friel, and J.C. Lavoie, Determinants of oxidant stress in extremely low birth weight premature infants. Free Radic. Biol. Med. 49 (2010) 1380-6.

[19] K.E. Joung, H.S. Kim, J. Lee, G.H. Shim, C.W. Choi, E.K. Kim, B.I. Kim, and J.H. Choi, Correlation of urinary inflammatory and oxidative stress markers in very low birth weight infants with subsequent development of bronchopulmonary dysplasia. Free Radic. Res. 45 (2011) 1024-32.

[20] K.B. Vera, D. Moore, E. Flack, M. Liske, and M. Summar, Significant Differences in Markers of Oxidant Injury between Idiopathic and Bronchopulmonary-Dysplasia-Associated Pulmonary Hypertension in Children. Pulm. Med. 2012 (2012) 301475.

[21] A. Fabiano, A.W. Gavilanes, L.J. Zimmermann, B.W. Kramer, P. Paolillo, G. Livolti, S. Picone, K. Bressan, and D. Gazzolo, The development of lung biochemical monitoring can play a key role in the early prediction of bronchopulmonary dysplasia. Acta Paediatr. 105 (2016) 535-41.

[22] S. Tokuriki, T. Okuno, G. Ohta, and Y. Ohshima, Carboxyhemoglobin Formation in Preterm Infants Is Related to the Subsequent Development of Bronchopulmonary Dysplasia. Dis. Markers 2015 (2015) 620921.

[23] J. Kuligowski, M. Aguar, D. Rook, I. Lliso, I. Torres-Cuevas, J. Escobar, G. Quintas, M. Brugada, A. Sanchez-Illana, J.B. van Goudoever, and M. Vento, Urinary Lipid Peroxidation Byproducts: Are They Relevant for Predicting Neonatal Morbidity in Preterm Infants? Antioxid. Redox Signal. 23 (2015) 178-84.

[24] M.A. Matthews, J.L. Aschner, A.R. Stark, P.E. Moore, J.C. Slaughter, S. Steele, A. Beller, G.L. Milne, O. Settles, O. Chorna, and N.L. Maitre, Increasing F2-isoprostanes in the first month after birth predicts poor respiratory and neurodevelopmental outcomes in very preterm infants. J. Perinatol. 36 (2016) 779-83.

[25] E.A. Abdel Ghany, W. Alsharany, A.A. Ali, E.R. Youness, and J.S. Hussein, Anti-oxidant profiles and markers of oxidative stress in preterm neonates. Paediatr Int Child Health 36 (2016) 134-40.

[26] C.C. Hsiao, J.C. Chang, L.Y. Tsao, R.C. Yang, H.N. Chen, C.H. Lee, C.Y. Lin, and Y.G. Tsai, Correlates of Elevated Interleukin-6 and 8-Hydroxy-2'-Deoxyguanosine Levels in Tracheal Aspirates from Very Low Birth Weight Infants Who Develop Bronchopulmonary Dysplasia. Pediatr. Neonatol. 58 (2017) 63-69.

[27] H.J. Rozycki, J. Bradley, and S. Karam, sRAGE Is Elevated in the Lungs of Premature Infants Receiving Mechanical Ventilation. Am. J. Perinatol. 34 (2017) 911-917.

[28] M. Laube, E. Amann, U. Uhlig, Y. Yang, H.W. Fuchs, M. Zemlin, J.C. Mercier, R.F. Maier, H.D. Hummler, S. Uhlig, and U.H. Thome, Inflammatory Mediators in Tracheal Aspirates of Preterm Infants Participating in a Randomized Trial of Inhaled Nitric Oxide. PLoS One 12 (2017) e0169352.

[29] J.T. Benjamin, R. van der Meer, J.C. Slaughter, S. Steele, E.J. Plosa, J.M. Sucre, P.E. Moore, J.L. Aschner, T.S. Blackwell, and L.R. Young, Inverse Relationship between Soluble RAGE and Risk for Bronchopulmonary Dysplasia. Am. J. Respir. Crit. Care Med. 197 (2018) 1083-1086.

[30] S. Bhunwal, K. Mukhopadhyay, S. Bhattacharya, P. Dey, and L.K. Dhaliwal, Bronchopulmonary Dysplasia in Preterm Neonates in a Level III Neonatal Unit in India. Indian Pediatr. 55 (2018) 211-215.

[31] H. Ozkan, N. Koksal, B.A. Dorum, F. Kocael, Y. Ozarda, C. Bozyigit, P. Dogan, I. Guney Varal, and O. Bagci, New-generation fish oil and olive oil lipid for prevention of oxidative damage in preterm infants: Single center clinical trial at university hospital in Turkey. Pediatr. Int. 61 (2019) 388-392.

[32] H.Y. Yildizdas, B. Poyraz, G. Atli, Y. Sertdemir, K. Mert, F. Ozlu, and M. Satar, Effects of two different lipid emulsions on antioxidant status, lipid peroxidation and parenteral nutrition- related cholestasis in premature babies, a randomized-controlled study. Pediatr. Neonatol. 60 (2019) 359-367.

[33] H. Go, H. Ohto, K.E. Nollet, K. Sato, K. Miyazaki, H. Maeda, H. Ichikawa, M. Chishiki, N. Kashiwabara, Y. Kume, K. Ogasawara, M. Sato, and M. Hosoya, Biomarker Potential of the Soluble Receptor for Advanced Glycation End Products to Predict Bronchopulmonary Dysplasia in Premature Newborns. Front Pediatr 9 (2021) 649526.

[34] C.C. Hsiao, C.H. Lee, R.C. Yang, J.Y. Chen, T.C. Su, Y.J. Chang, C.Y. Lin, and Y.G. Tsai, Heat Shock Protein-70 Levels Are Associated With a State of Oxidative Damage in the Development of Bronchopulmonary Dysplasia. Front Pediatr 9 (2021) 616452.

[35] X. Cui, and J. Fu, Urinary biomarkers for the early prediction of bronchopulmonary dysplasia in preterm infants: A pilot study. Front Pediatr 10 (2022) 959513.

[36] R. Urs, R. Ni Chin, N. Hemy, A.C. Wilson, J.J. Pillow, G.L. Hall, and S.J. Simpson, Elevated leukotriene B4 and 8-isoprostane in exhaled breath condensate from preterm-born infants. BMC Pediatr. 23 (2023) 386.

[37] N.A. Liyakat, P. Kumar, and V. Sundaram, Room air versus 100% oxygen for delivery room resuscitation of preterm neonates in low resource settings: A randomised, blinded, controlled trial. J. Paediatr. Child Health 59 (2023) 794-801.
